# A single dose of purified human antibody from transchromosomic bovines mitigates aerosolized Venezuelan equine encephalitis virus disease in cynomolgus macaques

**DOI:** 10.1371/journal.pone.0347864

**Published:** 2026-04-27

**Authors:** Chengqun Sun, Christina Gardner, Long Kwan Metthew Lam, Jeneveve Lundy, Katherine O’Malley, Thomas Luke, Kanakatte Raviprakash, Amy L. Hartman, Nicholas A. Crossland, Hua Wu, Jin-an Jiao, Christoph Bausch, Eddie Sullivan, Douglas S. Reed, William B. Klimstra

**Affiliations:** 1 University of Pittsburgh Center for Vaccine Research, Pittsburgh, Pennsylvania, United States of America; 2 University of Pittsburgh School of Medicine, Department of Immunology, Pittsburgh, Pennsylvania, United States of America; 3 Current address: United States Army Research Institute of Infectious Disease, Virology Division, Frederick, Maryland, United States of America; 4 Naval Medical Research Center Department of Viral and Rickettsial Diseases, Silver Spring, Maryland, United States of America; 5 University of Pittsburgh Graduate School of Public Health, Department of Infectious Diseases and Microbiology, Pittsburgh, Pennsylvania, United States of America; 6 National Emerging Infectious Diseases Laboratories, Comparative Pathology Laboratory, Boston University, Boston, Massachusetts, United States of America; 7 Boston University Chobanian and Avedisian School of Medicine, Department of Pathology and Laboratory Medicine, Boston, Massachusetts, United States of America; 8 S.A.B. Biotherapeutics, Inc, Miami, Florida, United States of America; Universidad Autónoma de San Luis Potosi, MEXICO

## Abstract

Venezuelan equine encephalitis virus (VEEV) is a mosquito-borne RNA virus that causes low mortality but high morbidity in humans. In addition to natural outbreaks, there is potential for exposure to VEEV via aerosolized virus particles. Currently there are no FDA-licensed vaccines or antiviral therapies for VEEV. We previously demonstrated that high titer anti-VEEV human antibody preparations derived from the plasma of hyperimmunized transchromosomic (Tc) bovines provided significant protection to mice from subcutaneous and aerosol challenge with virulent VEEV (Gardner et al., *J. Virol.*, 91:e00226-17, 2017). In the current studies we utilized the cynomolgus macaque primate model of VEEV infection, challenging some treatment groups with either a IA/B clade virus or a IC clade virus, each derived from a cDNA clone. Animals were treated with liquid or lyophilized transchromosomic bovine polyclonal antibody (TcpAb) preparations via intravenous (IV) or intranasal (IN) routes. Single dose prophylactic (24 hr before challenge) treatment with the liquid TcpAb given IV yielded significant reduction in the magnitude and/or extent of febrile responses and eliminated or greatly reduced serum infectious viremia *versus* control after aerosol challenge with VEEV regardless of subtype. Therapeutic (24 hr after challenge) treatment showed similar results with the IA/B virus. However, IN treatment alone with lyophilized TcpAb was ineffective against the IC virus. These results indicate that IV treatment with the TcpAb is effective in reducing VEEV disease severity in non-human primates and may be a useful single treatment strategy for infections with this emerging virus.

## Introduction

Venezuelan equine encephalitis virus (VEEV) is an enveloped, single-stranded, positive sense RNA virus that causes epizootic and epidemic outbreaks in equines and humans [1]. In humans, VEEV is rarely fatal (<1% of infections) but causes a biphasic febrile illness where most patients experience acute debilitation with ~10% of suffering from neurological disease and permanent neurological sequalae [2, 3]. During the Cold War, both the United States and the Soviet Union developed VEEV as a potential biological warfare agent [4–6], and there are currently no FDA-licensed vaccines or antiviral therapies available to combat VEEV infection. Due to the lack of prevention and treatment strategies, development of new vaccines and antiviral therapies is of high importance. Passive antibody therapy is considered a promising approach to treating many infectious viral and bacterial diseases including rapidly emerging viruses such as SARS-CoV-2 (reviewed in [7–10]. Only one study has examined anti-VEEV antibody use in macaques demonstrating that a monoclonal antibody had partially protective effects on viremia and fever when given 24 or more hours post-infection [11].

The transchromosomic (Tc) bovine model is a novel approach to rapidly generate neutralizing polyclonal antibody preparations. Tc bovines have been engineered to possess a human artificial chromosome expressing the human antibody heavy chain and kappa light chain. These animals also are deleted for bovine heavy chain genes and lambda cluster light chain genes (*IGHM*^*-/-*^ *IGHML1*^*-/-*^ *IGL*^*-/-*^) [12–14]. The Tc bovines produce three kinds of IgG antibodies: human IgG, chimeric IgG (containing human heavy chain and bovine kappa chain), trans-class switched bovine IgG as well as IgA. During the purification process, chimeric and bovine IgG are separated from the fully human IgG, resulting in a fully-human polyclonal IgG antibody preparation. Tc bovines can produce as much as 300 grams/animal/month of human IgG, allowing production of highly concentrated antibody preparations in a very short time frame for therapeutic use [12]. These preparations have been safely and effectively used in pre-clinical studies for treatment of several infectious diseases [15–22]. We recently demonstrated that passive immunotherapy using a polyclonal TcpAb preparation was protective in mice after subcutaneous or aerosol challenge with VEEV [23].

Demonstration of safety and efficacy in two animal models will likely be required for eventual FDA licensing of this type of therapeutic treatment for an emerging virus infection [24, 25]. Our studies investigated the protective efficacy of administering a candidate polyclonal TcpAb, SAB-131, via various formulations (liquid or lyophilized) and routes (intravenous, IV or intranasal, IN) against aerosol challenge of cDNA-derived clones of VEEV from the IA/B clade (Trinidad Donkey strain) or the IC clade (INH9813-K3E epizootic strain). While macaques receiving control antibody showed febrile responses and serum viremia, IV anti-VEEV TcpAb treatment reduced fever duration and viremia. However, the lyophilized formulation lacked efficacy on its own and did not augment the efficacy of the IV treatment. Overall, IV delivery of TcpAB in a liquid formulation is effective against VEEV in a non-human primate model of aerosol infection, suggesting potential utility as an anti-VEEV therapeutic. Further work on improving delivery of lyophilized powder to the respiratory tract will need to be conducted.

## Materials and methods

### Ethics statement

All animal studies were conducted in accordance with the recommendations outlined in the Guide for the Care and use of Laboratory Animals of the National Research Council. Procedures were approved by the Institutional Animal Care and Use Committee (IACUC) of the University of Pittsburgh and SAB Biotherapeutics in compliance with all applicable Federal regulations governing the protection of animals in research.

### Cultured cells

BHK-21 cells (ATCC CCL-10) were maintained in RPMI 1640 media supplemented with 10% donor bovine serum (DBS) and 10% tryptose phosphate broth. Vero cells (ATCC CCL-81) were maintained in DMEM supplemented with 10% fetal bovine serum (FBS). All media for cell lines also supplemented with penicillin (100 U/mL), streptomycin (0.05 mg/mL) and L-glutamine (0.05 mg/mL). All cells were incubated at 37 °C with 5% CO_2_.

### Viruses and replicons

Construction of the Venezuelan equine encephalitis virus (VEEV) Trinidad Donkey strain (V3000) and the INH9813-K3E cDNA clones have been previously described [26–28]. The K3E version of the cDNA clone of INH9813 is used as wild type as described [27]. The construction of the cDNA clones for a VEEV TrD eGFP expressing replicon genome and packing helpers has been described [27]. Virus and replicon stocks were generated from cDNA clones by *in vitro* transcription (IVT) from linearized cDNA plasmid templates as previously described [28, 29]. Briefly, IVT kit (mMessage mMachine, Ambion) was used to generate infectious, capped viral RNA genomes that were electroporated BHK cells. The supernatant was clarified by centrifugation at 18–24 hr post-electroporation, and used for high MOI infection of Vero cells followed by 24 hr incubation, clarification and separating on a discontinuous 20%/60% sucrose gradient at 24,000 rpm followed by similar pelleting of virus over a 20% sucrose cushion. Virus pellets were resuspended in HEPES buffered Opti-MEM (Gibco) with 1% FBS, and single-use aliquots were stored at −80 °C. Virus stock titers were determined by standard plaque assay or infectious unit assay on BHK-21 cells [23, 27].

### Production of SAB-131 antibody preparation

A detailed description of the preparation of inactivated VEEV antigen from an attenuated VEEV Trinidad Donkey strain mutant, the induction of anti-VEEV neutralizing immunoglobulins in human immunoglobulin transchromosomic bovines and production and purification of human immunoglobulin preparations has been presented [23]. The control Dengue virus specific Tc bovine antibody preparation was also produced as described [23].

### Infectivity reduction/plaque reduction neutralization test (IRNT/PRNT)

VEEV TrD eGFP propagation-incompetent replicons [23] or INH9813K3E viruses were diluted in virus diluent (PBS-1% DBS) and reacted with bovine or mouse serum for 1 hr at 37°C before being used to infect 24-well plates of Vero cells for 1 hr at 37°C as described [23]. For IRNT, culture media was added to the cells and the cells were incubated for 24 hr before being fixed with 4% PFA. GFP-expressing (infected) cells were then quantified on an Olympus CKX41 inverted fluorescence microscope. For PRNT, after infection, immunodiffusion agarose was added, plates were incubated for 48 hr and then stained with neutral red and plaques were counted on a light box as previously described [23].

### Virus stocks

Virus stocks were validated for virulence prior to macaque studies by aerosol infection (~100 LD_50_) of CD-1 mice, which were housed under specific pathogen-free conditions. 10 mice were used to test virus stock virulence. All mice were weighed daily and monitored by trained veterinary or research staffs for morbidity and mortality, which was increased to twice daily upon onset of clinical signs of disease. Mice losing more than 35% of weight or showing severe signs of disease (unable to move or feed, paralysis, or moribund) were euthanized promptly via isoflurane overdose. Mice surviving by 14 dpi (end of experiment) were sacrificed. Only stocks that caused 100% mortality in the mice were used for macaque experiments.

### Macaque study design and husbandry

**Fig 1:**
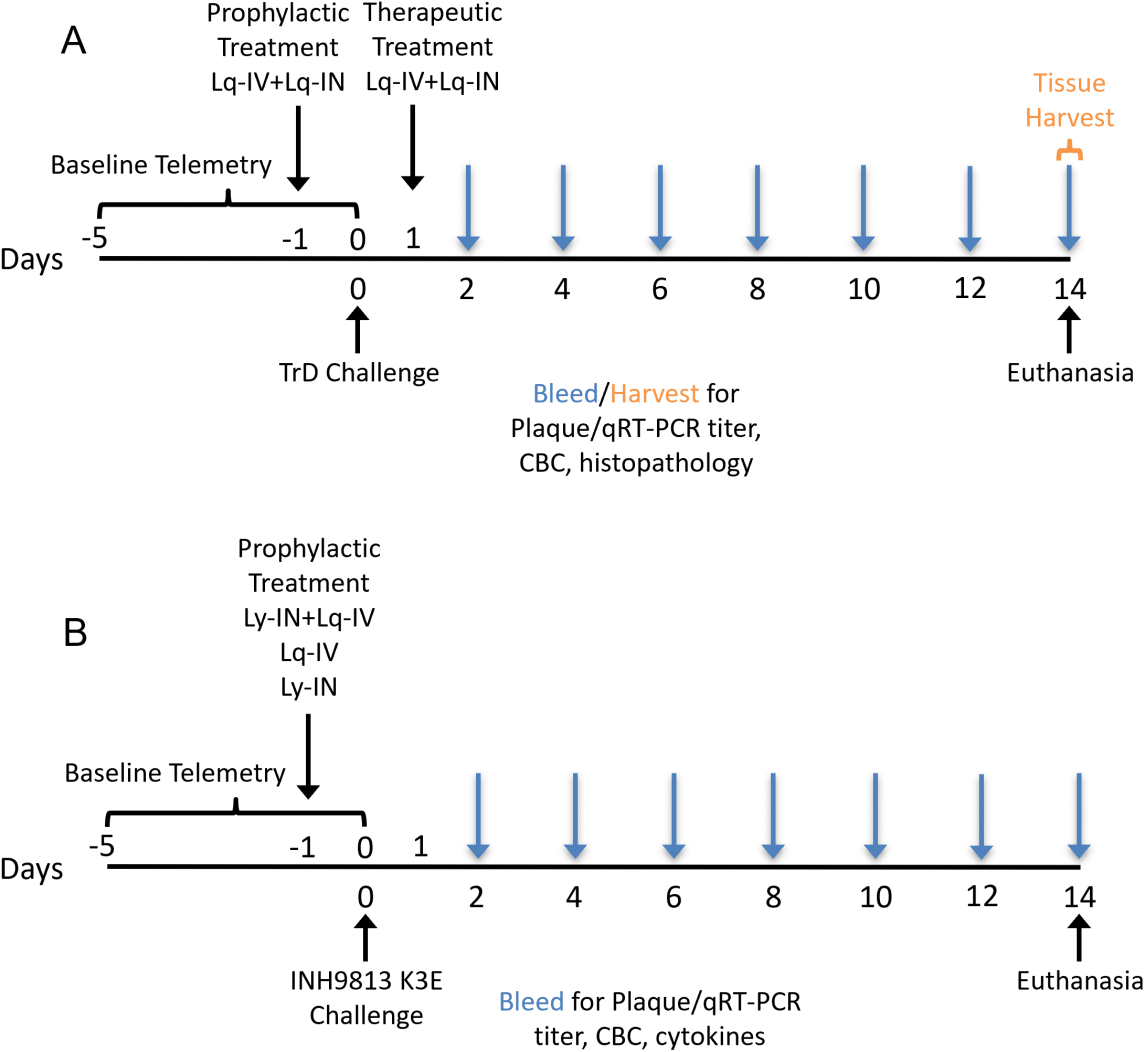
Timeline of macaque baseline telemetry. SAB-131 treatment, bleeding and tissue harvesting for cohort 1 (A) and cohort 2 (B) animals.

Cynomolgus macaques ranged from 2–9 years of age and included both male and female animals were used. Prior to use, the macaques were verified to be serologically negative for Herpes virus B, SIV, STLV, and SRV and free of neutralization activity against VEEV (<1:20 in PRNT_50_). Timeline and treatment for macaque studies are shown in figure 1. VEEV is non-lethal in this cynomolgus macaque model [28]. Macaques were clinically monitored and assessed twice daily, with temperature monitored by telemetry. Clinical scores parameters comprised of neurological score, activity score, and temperature score were previously reported and approved by the University of Pittsburgh IACUC committee [30]. Detailed scoring parameters are presented in S1 table. Animals were promptly euthanized if they met any of these criteria: a composite score of 14 or above, reaching the highest score in any of the three scoring categories, greater than 20% weight loss, unable to ambulate for food and water, or seizure lasting more than 30 min.

Environmental room parameters were maintained in accordance with applicable federal regulations and recommendations of the Guide for the Care and Use of Laboratory Animals of 12 hr light cycle of light: dark, 64–84 °F temperature, and 30–70% relative humidity. Macaques were fed a species-appropriate commercially available diet twice daily by veterinary or research staffs trained by veterinarians. Additional food items approved by university veterinarian were given for environmental enrichment (e.g., fruits, vegetables, cereal, popcorn) and were provided by a variety of methods, including but not limited to puzzle feeders and foraging boards. Clean, potable drinking water was provided ad libitum via an automated water delivery system. Other provided enrichment included foraging opportunities, human interactions, stimulation of all five senses, and means to control their environment though manipulations and cognition stimulating activities. Animals were socially housed whenever possible. An enhanced enrichment plan was followed due to a social housing exemption following infection in the Regional Biocontainment Laboratory.

### General animal procedures

Macaques were implanted with telemetry devices as described below. As needed, macaques were sedated for phlebotomy with 10 mg/kg ketamine administered via intramuscular injection using a safety needle and blood collected from the femoral or saphenous vein. For euthanasia, macaques were sedated with 20 mg/kg ketamine followed by injection of sodium nitroprusside mixed with 12 mL of NaCl, followed by 200 mg/kg of intravenous Beuthanasia-D-Special. Once euthanasia was confirmed, macaques were perfused via the left ventricle with saline. Tissues were inactivated in 10% neutral buffered formalin. Paraffin-embedded tissues were cut, mounted on slides, and stained with H&E for pathology interpretation.

Details for treatment for each macaque were listed in Table 2. Treatment groups were either infused in the saphenous vein with ~100 mg/kg of liquid anti-VEEV TcpAb or anti-Dengue virus control Tc Ab, were treated intranasally with 37.5 mg/kg of liquid anti-VEEV or anti-DENV TcpAb per nostril, or were treated with 40 mg of lyophilized anti-VEEV or anti-DENV TcpAb either 24 hours before or 24 hours after aerosol challenge with ~1 x 10^7^ plaque forming units (PFU) of VEEV INH9813-K3E or TrD strains. This aerosol dose of either TrD or INH9813-K3E was previously determined to cause a biphasic febrile disease in similar cynomolgus macaques [28, 31]. The antibody dose was chosen based on previous reports of efficacious TcpAb doses in non-human primates [19, 21].

### Telemetry implant surgery

Each macaque was equipped with a PhysioTel Digital radiotelemetry implant (Data Sciences International, DSI, St. Paul, MN) capable of continuously transmitting EEG, ICP, and temperature. Prior to implantation surgery, each macaque was anesthetized by injection of ketamine hydrochloride (20 mg/kg) and atropine (0.4 mg/kg); once anesthesia was confirmed, macaques were maintained on ~1.5% isoflurane gas anesthesia for the duration of surgery. Each macaque received an IV catheter in the greater saphenous vein for 3% normal saline, a tracheal tube for intubation, with continuous pulse oximetry and rectal thermometry for vital sign monitoring. Prior to draping and fixture into a stereotaxic apparatus, the head, neck, and upper back of each macaque was shaved and scrubbed in triplicate with betadine and chlorhexidine. Surgical implantation of the implant was performed as previously described [32]. Post-surgery, macaques were given analgesia and observed until recovered. At least 2 weeks after surgery, macaques were transferred to the ABSL-3 facility.

### Aerosol exposure

Aerosol exposures were performed under the control of the Aero3G aerosol management platform (Biaera Technologies, Hagerstown, MD) as previously described [28]. Macaques were anesthetized with 6 mg/kg Telazol® (Tiletamine HCl / Zolazepam HCl); once anesthesia was confirmed the macaque was weighed, bled, and transported to the Aerobiology suite using a mobile transport cart. The macaque was then transferred from the cart into a class III biological safety cabinet and the macaque’s head was placed inside a head-only exposure chamber. Jacketed External Telemetry Respiratory Inductive Plethysmography (JET-RIP; DSI) belts were placed around the upper abdomen and chest of the macaque and calibrated to a pneumotach. This allowed monitoring and recording of respiratory function during the exposure via the Ponemah software platform (DSI) during the aerosol [28]. Exposures were either 10 minutes in duration or accumulated tidal volume-based using the JET-RIP interface with the Biaera software. Aerosols were generated using an Aerogen Solo vibrating mesh nebulizer (Aerogen, Chicago, IL) as previously described [33]. To determine inhaled dose, an all-glass impinger (AGI; Ace Glass, Vineland, NJ) was attached to the chamber at operated at 6 lpm, −6 to −15 psi. Particle size was measured once during each exposure at 5 minutes using an Aerodynamic Particle Sizer (TSI, Shoreview, MN). Virus concentration in nebulizer and AGI samples was assessed by plaque assay; inhaled dose was calculated as the product of aerosol concentration of the virus and the accumulated volume of inhaled air [34]. All animal experiments were conducted at ABSL-3 in accordance with AAALAC-approved institutional guidelines for animal care and used approved by the University of Pittsburgh IACUC committee.

### Temperature data acquisition and analysis

Signals from DSI implants were transmitted to TRX-1 receivers connective via a communication link controller (CLC) to a computer running Ponemah v5.2 or 5.3 (DSI). Closed circuit cameras (Axis Model No. M-1145) were positioned to continuously record macaque behavior including neurological abnormalities such as seizures and sleep disruption. Camera video was recorded through a Ponemah interface with MediaRecorder software (Noldus Information Technology, Leesburg, VA). Telemetry and video data was collected continuously from a baseline period (at least two days preceding aerosol challenge) until necropsy. At least once daily, data acquisition was stopped, data was transferred to a network server, and then acquisition was restarted.

Temperature data collected via radiotelemetry was exported from Ponemah as 15-minute averages into an Excel spreadsheet. Data were checked for missing or erroneous data points (body temperature <27 °C or >43 °C) and analyzed using an ARIMA model in MATLAB R2019A (MathWorks, Natick, MA). Prior to challenge, for each macaque the baseline temperature data were used to generate hourly temperature ranges for use in clinical scoring to determine significant deviations in temperature. Because the EEG/ICP implants were subcutaneous on the upper back of the macaque, temperature readings were lower than would be expected for core body temperatures but still exhibited 1–2 °C diurnal variation prior to challenge. The code used is available on GitHub at https://github.com/ReedLabatPitt/Reed-Lab-Code-Library.

### Virus titration

cDNA was synthesized from viral RNA using Invitrogen™ M-MLV Reverse Transcriptase (Fisher Scientific) in 20 μL reactions with either 100ng of total RNA from tissue-type samples or 5 μL of total RNA prep (1/8 of total RNA isolation volume) from liquid-type samples such as serum plasma. RNA template was added to 2 pmol of gene specific T7-linked RT primer (VEEV INH9813 nsP2 - GCGTAATACGACTCACTATAGTCTTCTGTTCACAGGTACTAGAT) and 10 mM dNTP mix and the mixture heated to 65°C for 5 minutes before rapidly cooling on ice to promote primer binding to template. After cooling for 60 seconds, buffer, 0.1 M DTT, Rnase Inhibitor and M-MLV enzyme provided by the manufacturer were mixed with prepared template/primer complex and incubated at 37 °C for 50 minutes; samples were then heated to 70 °C to inactivate M-MLV transcriptase.

Quantitative PCR of amplified cDNA was performed with Applied Biosystems™ TaqMan™ Fast Universal PCR Master Mix (2X) (Fisher Scientific) on an Applied Biosystems™ QuantStudio 6 Flex Real-Time PCR System. A gene-specific primer, gene specific probe and a T7 primer combination was employed to enhance specificity of target. The primers are as follows: VEEV TrD/INH9813 nsP2 primer (18 μM)– CCGGAAGAGTCTATGACATGAA; T7 promoter (18 μM) – GCGTAATACGACTCACTATA; Probe (5 μM) – 56-FAM/CTGGCACGCTGCGCAATTATGATC/3BHQ-1. Primers and probe were added to 5 μL of amplified cDNA in 20 μL reaction volume. The RT-qPCR system was run on a fast-cycling protocol: 95 °C, 20 seconds hold; 45 cycles between 95 °C, 3 seconds and 60 °C, 20 seconds. Data were collected and analyzed on Applied Biosystems™ Quant Studio Real-Time PCR Software; graphs and statistics were generated with Prism by GraphPad Software, LLC. Serum viremia was determined by standard alphavirus plaque assay on BHK cells (as described above) at each blood sampling time. A conservative limit of detection was established in cohort 1 and cohort 2 assays, using RNA derived from blood of multiple uninfected macaques, so the detection of viral RNA is consistent with the presence of some form of virus genomic RNA.

### Whole Blood Processing

Animals were sedated for sampling after infection by administration of 10 mg/kg ketamine via intramuscular injection. Once sedated, 2−3 mL of blood was drawn from either the right or left femoral vein into an EDTA tube. Plasma was frozen for serologic, immunologic and virologic assays. CBC analysis was performed using the Abaxis HM5 hematology analyzer. Blood chemistry analysis was performed using the Comprehensive Diagnostic Panel rotor (Abaxis 500−0038) on an Abaxis VS2 chemistry analyzer.

### Cytokine analysis

Cytokine analysis was performed on plasma using a Luminex bead array NHP custom 30-plex kit (EPX300-40044-901, Invitrogen). Analytes measured were interleukin IL-1beta, IL-1ra, IL-2, IL-4, IL-5, IL-6, IL-7, IL-8, IL-10, IL-12p40, IL-13, IL-15, IL-17A, Eotaxin, G-CSF, GM-CSF, IFN-gamma, IP-10, MCP-1, MIP-1alpha, MIP-1beta, TNF-alpha, SDF-1alpha, MIG, I-TAC, BLC, IL-23, IL-18, CD40-L, and IFN-alpha.

### Histopathology Analysis

Macaques from cohort 1 were necropsied at 28 dpi for collection of distinct anatomical compartments of the brain (olfactory bulb, frontal, parietal, temporal, & occipital lobes, and the cerebellum) and spleen for histopathologic analysis. Samples were fixed in 10% neutral buffered formalin for a minimum of 30 days before removal from biocontainment and then processed routinely as formalin fixed paraffin embedded (FFPE) blocks for generation of hematoxylin & eosin (H&E) differentially stained slides. The study pathologist (N.A.C.) was initially blinded for initial ordinal scoring and qualitative descriptions, upon which was unblinded and results organized to generate finalized pathology results. Meningeal infiltrates, perivascular infiltrates, and gliosis were scored as such: 0-not observed, 1-minimal, 2-mild, 3-moderate, 4-marked, 5-severe. Represented H&E images were acquired using a PhenoImager whole slide scanner (Akoya Biosciences, Marlborough, MA) for figure preparation.

### Statistical analysis

Statistical analyses comparing treatment group averages included evaluation of the significance of maximum fever deviation from baseline on each day, fever duration, and fever severity (fever-hours), viremia levels, blood cell population analyses and serum cytokine levels. Statistical tests including t-test, ANOVA, and Log-Rank were performed on the data sets. Analyses were performed with Microsoft Excel or GraphPad PRISM software or other methods as described for individual data sets. For cytokine and blood chemistry data, two-way ANOVA was performed. Plaque and qRT-PCR titers in individual animals were compared at each blood collection time point by unpaired Student’s t-test. Telemetric temperature measurements were compared by two-way ANOVA or Brown-Forsythe ANOVA as indicated.

## Results

### Treatments and animals

To elicit human anti-VEEV antibodies, Tc bovines were immunized up to five times with inactivated VEEV nt5 virus (described in [23]). Sera from Tc bovines were pooled, purified and concentrated and TcpAb preparations were prepared as described in materials and methods (designated as SAB-131). Lyophilized SAB-131 preparations were reconstituted in sterile water (Gibco) to a concentration of 1 mg/mL and liquid SAB-131 was diluted to 1 mg/mL prior to neutralization assays. Both the liquid and the reconstituted lyophilized TcpAb preparations (SAB-131) exhibited high neutralization capacity against the VEEV TrD and INH9813-K3E strains, and the negative control preparation failed to neutralize (Table 1).

**Table 1:** Neutralization titers (IRNT80 or PRNT801) for SAB TcpAb preparations.

Preparation	Dilution endpoint for TrD IRNT_80_	Dilution endpoint for INH9813 PRNT_80_	Description
SAB-131 liquid	>1:40,960	1:20,480	Anti-VEEV Ab preparation
SAB-131 lyophilized^2^	>1:40,960	1:20,480	Anti-VEEV Ab preparation
DENV TcpAb liquid	<1:20	<1:20	Irrelevant Ab preparation
DENV TcpAb lyophilized^2^	<1:20	<1:20	Irrelevant Ab preparation
ATCC anti-VEEV mouse ascites	1:2,560	1:2,560	Immunized mouse ascites fluid (positive control)

1 Infection reduction neutralization assay. Dilution endpoint for 80 percent neutralization of Vero cell infectivity of VEEV TrD GFP-expressing replicon particles. Plaque reduction neutralization assay as with IRNT except reduction of Vero cell INH9813-K3E plaques in a standard plaque assay.

2 Lyophilized TcpAb was reconstituted in sterile water to a concentration of 1 mg/ml to equal the beginning concentration of TcpAb in the liquid formulation.

Macaques determined to be free of anti-VEEV reactivity by serum neutralization assay were used in experiments. Clinical scores and telemetric recording of body temperature were used to monitor disease progression upon VEEV infection. Protection experiments reported here were comprised of two cohorts (Fig 1 and Table 2): Cohort 1 examined protection against aerosol infection of VEEV TrD, an epizootic IA/B clade virus. This cohort contains a prophylactic and a therapeutic arm. In the prophylactic group, macaques were infused intravenously with 100 mg/kg of anti-VEEV in liquid formulation (Lq-IV) and 75 mg/kg of reconstituted antibody in 1mL as direct deposition split between the nostrils (Lq-IN) 24-hr prior to infection. Two control macaques received anti-dengue virus (DENV) antibodies prophylactically. In the therapeutic arm, macaques were challenged with VEEV first and then received anti-VEEV antibody preparations at 1 day post infection (dpi), and one control macaque received anti-DENV antibodies therapeutically.

**Table 2:** Characteristics and study design for Cohort 1 and Cohort 2 animals.

Animal Number	Sex	TcpAb treatment	Ab amount	Route (Group)	SAB-31 timing to challenge	Virus Challenge	Targeted Aerosol Dose (Log_10_ PFU)
Cohort 1 (animal ID)							
1 (171)	M	Anti-DENV liquid	100 mg/kg IV 75 mg/kg liquid IN	Lq-IV + Lq-IN	24 hours pre	TrD (IA/B)	7
2 (172)	M	Anti-DENV liquid	100 mg/kg IV 75 mg/kg liquid IN	Lq-IV + Lq-IN	24 hours pre	TrD (IA/B)	7
3 (173)	M	Anti-DENV liquid	100 mg/kg IV 75 mg/kg liquid IN	Lq-IV + Lq-IN	24 hours post	TrD (IA/B)	7
4 (165)	M	SAB-131 liquid	100 mg/kg IV 75 mg/kg IN	Lq-IV + Lq-IN	24 hours pre	TrD (IA/B)	7
5 (169)	M	SAB-131 liquid	100 mg/kg IV 75 mg/kg IN	Lq-IV + Lq-IN	24 hours pre	TrD (IA/B)	7
6 (170)	M	SAB-131 liquid	100 mg/kg IV 75 mg/kg IN	Lq-IV + Lq-IN	24 hours pre	TrD (IA/B)	7
7 (166)	M	SAB-131 liquid	100 mg/kg IV 75 mg/kg IN	Lq-IV + Lq-IN	24 hours post	TrD (IA/B)	7
8 (167)	M	SAB-131 liquid	100 mg/kg IV 75 mg/kg IN	Lq-IV + Lq-IN	24 hours post	TrD (IA/B)	7
9 (168)	M	SAB-131 liquid	100 mg/kg IV 75 mg/kg IN	Lq-IV + Lq-IN	24 hours post	TrD (IA/B)	7
Cohort 2 (animal ID)							
10 (101)	F	Anti-DENV lyophilized	40 mg/nostril	Ly-IN	24 hours pre	INH9813-K3E (IC)	7
11 (99)	M	Anti-DENV lyophilized	40 mg/nostril	Ly-IN	24 hours pre	INH9813-K3E (IC)	7
12 (109)	F	Anti-DENV lyophilized	40 mg/nostril	Ly-IN	24 hours pre	INH9813-K3E (IC)	7
13 (97)^1^	M	SAB-131 lyophilized	20 mg/nostril	Ly-IN	24 hours pre	INH9813-K3E (IC)	7
14 (98)	M	SAB-131 lyophilized	40 mg/nostril	Ly-IN	24 hours pre	INH9813-K3E (IC)	7
15 (104)	F	SAB-131 lyophilized	40 mg/nostril	Ly-IN	24 hours pre	INH9813-K3E (IC)	7
16 (110)	F	SAB-131 lyophilized	40 mg/nostril	Ly-IN	24 hours pre	INH9813-K3E (IC)	7
17 (102)	M	SAB-131 lyophilized	40 mg/nostril	Ly-IN	24 hours pre	INH9813-K3E (IC)	7
18 (108)	F	SAB-131 liquid	100 mg/kg IV	Lq-IV	24 hours pre	INH9813-K3E (IC)	7
19 (103)	M	SAB-131 liquid	100 mg/kg IV	Lq-IV	24 hours pre	INH9813-K3E (IC)	7
20 (106)	F	SAB-131 liquid	100 mg/kg IV	Lq-IV	24 hours pre	INH9813-K3E (IC)	7
21 (96)	M	SAB-131 lyophilized SAB-131 liquid	40 mg/nostril 100 mg/kg IV	Ly-IN+Lq-IV	24 hours pre	INH9813-K3E (IC)	7
22 (100)	F	SAB-131 lyophilized SAB-131 liquid	40 mg/nostril 100 mg/kg IV	Ly-IN+Lq-IV	24 hours pre	INH9813-K3E (IC)	7
23 (107)	F	SAB-131 lyophilized SAB-131 liquid	40 mg/nostril 100 mg/kg IV	Ly-IN+Lq-IV	24 hours pre	INH9813-K3E (IC)	7

Animal received half the intended dose of lyophilized SAB-131

Cohort 2 examined the efficacy of various formulations of prophylactic SAB-131 treatment against aerosol infection of INH9813-K3E, an epizootic IC clade VEEV (Fig 1B and Table 2). In addition to Lq-IV delivery as in cohort 1, we delivered lyophilized TcpAb intranasally (Ly-IN) to the nostrils via a compression bulb-operated TrivAir dry drug delivery device. The target dose was 40 mg; however, 1 out of the 6 macaques mistakenly received only 20 mg. We also examined the effects of combined formulations, where macaques received both Lq-IV and Ly-IN (Lq-IV + Ly-IN) of SAB-131. Control macaques received 40 mg anti-DENV TcpAb through the IN route (Ly-IN) (Table 2). In preliminary experiments based on ejection of the lyophilized powder after a single bulb compression, we determined that 40 mg was the maximum quantity that could be reliably delivered by the TrivAir device.

### Telemetry for temperature

**Fig 2:**
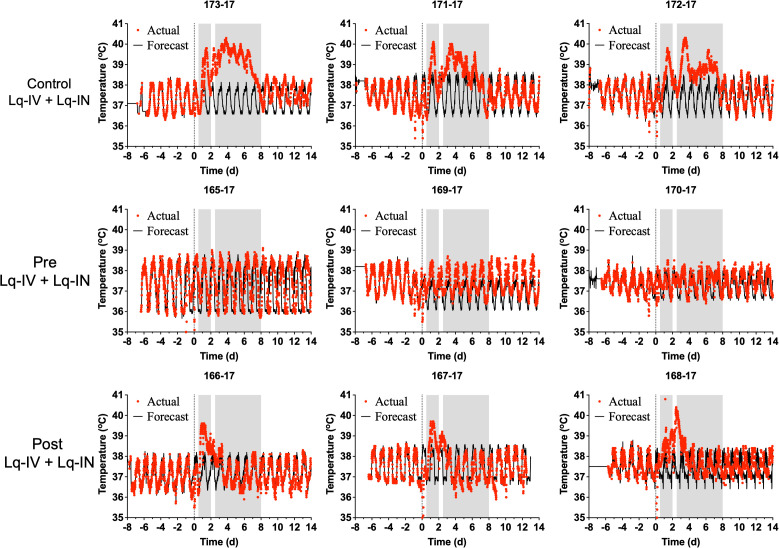
Febrile responses for cohort 1. Actual (red lines) and forecasted (black lines) temperature telemetry data from cohort 1 cynomolgus macaques challenged with VEEV TrD and either treated with control liquid TcpAb (row A) or treated with liquid anti-VEEV TcpAb 24 hours prior to infection (Pre) or 24 hours after infection (Post). Shaded areas represent the first and second febrile phases determined a previous study [28]. Forecast temperatures are calculated with NCSS-PASS Software based upon the at least 3 days of data recorded prior to challenge. The number above each temperature profile corresponds to the unique identification number of the macaque.

**Fig 3:**
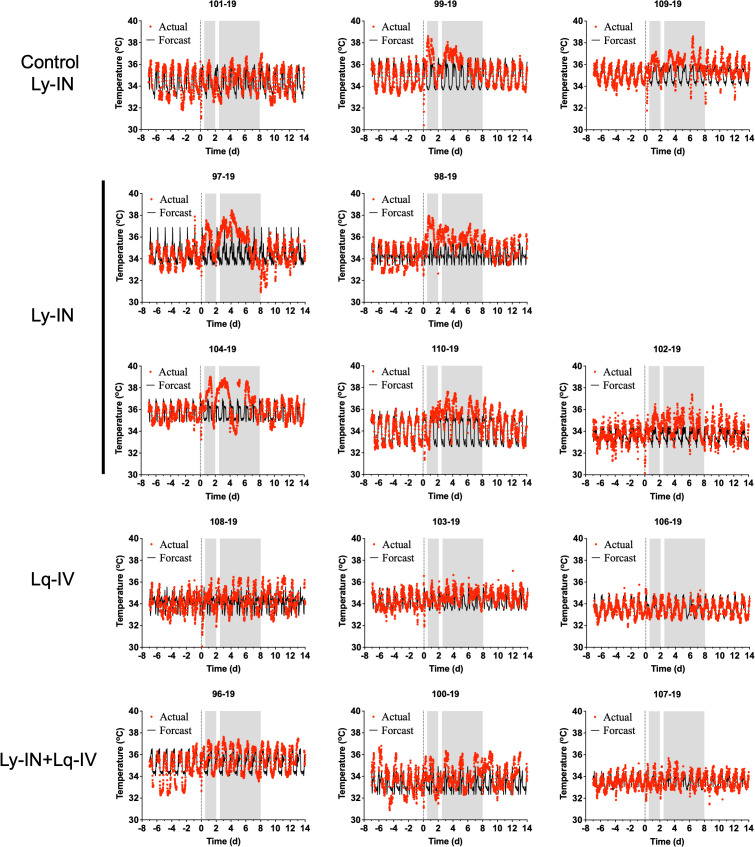
Febrile responses for cohort 2. Actual (red lines) and forecasted (black lines) temperature telemetry data from cohort 2 cynomolgus macaques challenged with VEEV INH9813-K3E and either treated 24 hours prior to challenge with control TcpAb (Control, n = 3), intranasal lyophilized SAB-131 (Ly-IN, n = 5), intravenous liquid SAB-131 (Lq-IV, n = 3), or intranasal lyophilized and intravenous liquid SAB-131 (Ly-IN+Lq-IV, n = 3). Shaded areas represent the first and second febrile phases determined a previous study [28]. Forecast temperatures are calculated with NCSS-PASS software based upon at least 3 days of data recorded prior to challenge. The number above each temperature profile corresponds to the unique identification number of the macaque.

**Fig 4:**
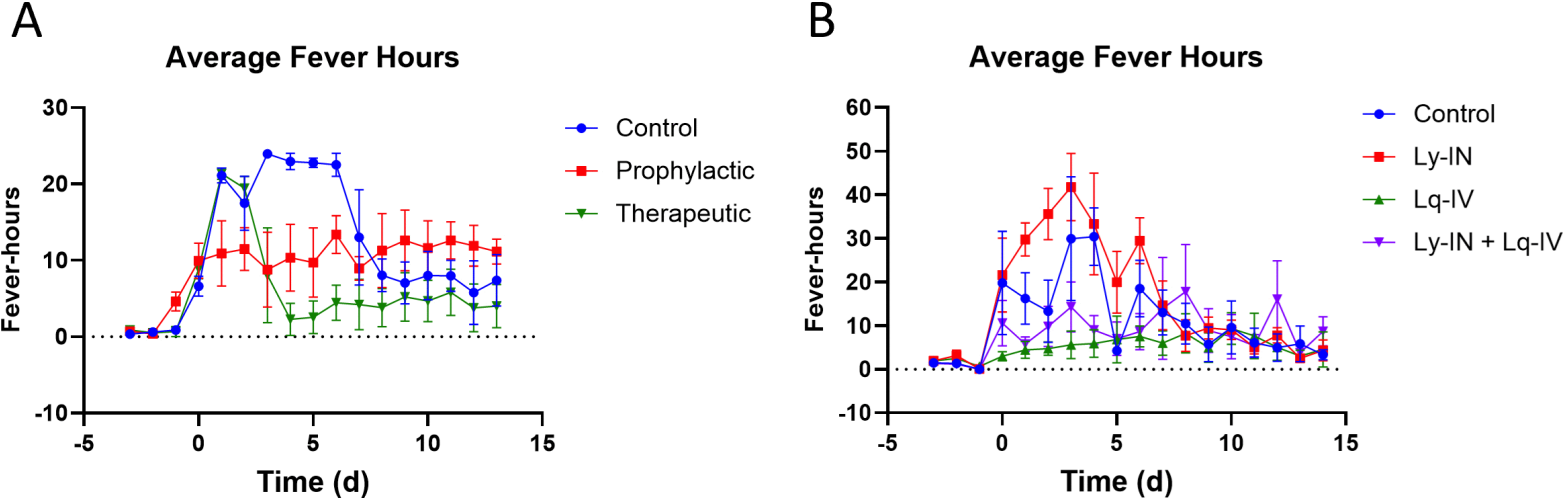
Fever severity in macaques. Averaged fever hours (differences between predicted and actual temperature) by day post challenge for cohort 1 (A) or cohort 2 **(B)** NHPs treated with control Abs or pre- or post-treated with anti-VEEV TcpAb. Mean and SEM are presented.

Because fever is a typical sign for VEEV infections in macaque, we monitored the body temperature of macaques via telemetry (Figs 2-4). Changes from predicted body temperature were quantified, including maximum deviation from baseline (max ΔT), fever duration, and fever severity (fever-hours; summation of significant residual elevations in body temperature divided by 4 to convert to hours). Results between treated and control groups were then compared to evaluate whether treatment reduced fever or hypothermia.

In cohort 1, control animals exhibited the typical biphasic fever profile associated with VEEV TrD aerosol infection with an initial fever spike between 0–2 dpi that resolved and a second spike starting between 3 and 4 dpi and resolving between 7 and 8 dpi (Fig 2). Antibody pre-treatment essentially eliminated both phases of the febrile response as compared with controls, although minor temperature elevations were present. However, the number of animals per group was too low to analyze most data for statistical significance (Fig 4A and Table 3). The maximum fever achieved was significantly lower for pre-treatment group (p < 0.01) than controls. With post-treatment, the initial phase of fever developed as with control animals but after treatment at 1 dpi, suppression of the second febrile phase was observed (Fig 4A). Post-treatment either suppressed the magnitude and duration of fever (Fig 2, animals 166−17 and 167−17) or the duration of febrile responses (Fig 2, animals 166−17, 167−17, and 168−17). Regardless of treatment group, antibody treatment reduced the fever duration by approximately 3 days. A comparison of overall fever severity for each treatment group (Table 3) showed a trend towards reduced fever duration and severity (fever-hours) in both treatment groups (p < 0.05).

**Table 3:** Mean values for macaque fever responses in control and treatment groups.

	Overall				First fever period				Second fever period			
Cohort 1	Max ΔT	Duration (hours)	Fever-Hours	Ave. Elev.	Max ΔT	Duration (hours)	Fever-Hours	Ave. Elev.	Max ΔT	Duration (hours)	Fever-Hours	Ave. Elev.
Control	2.9	184.7	261.0	1.4	1.9	14.7	17.7	1.1	1.8	120.2	193.4	1.6
Pre-treatment	1.2**	36.7**	25.7**	0.7*	0.9	4.0	3.1	0.7	1.1**	9.8	6.6	0.6
Post-treatment	2.9	79.7*	99.2*	1.2	2.8	29.6	45.3	1.5	1.2	30.4	38.3	1.2
	Overall				First fever period				Second fever period			
Cohort 2	Max ΔT	Duration (hours)	Fever-Hours	Ave. Elev.	Max ΔT	Duration (hours)	Fever-Hours	Ave. Elev.	Max ΔT	Duration (hours)	Fever-Hours	Ave. Elev.
Control	3.49	177.83	230.19	1.33	3.14	20.00	35.27	1.61	3.08	71.17	108.47	1.48
Ly-IN	3.98	208.75	338.78	1.64	3.35	29.10	57.06	1.93	3.79	84.35	164.49	1.96
Lq-IV	2.45	94.83	121.89	1.12	1.69	7.50	8.92	1.06	2.39	26.25	33.99	1.19
Ly-IN+ Lq-IV	3.1	182.83	252.58	1.28	2.03	13.08	14.69	1.15	2.86	45.50	61.42	1.31

*: p < 0.05

**: p < 0.01

Test group versus control.

In cohort 2, two of the three control macaques experienced two febrile periods after aerosol exposure to VEEV INH8813 K3E - one during 1−2 dpi and the second between 2.5−8 dpi (Fig 3). The third control (101−19) had only mild temperature elevations in those two periods but also developed a mild hypothermia in the recovery period (Fig 3, 8–14-14 dpi). This unusual fever profile has previously been observed by our group [28], and is likely due to the outbred nature of the macaques used in the studies. In the Ly-IN group, all five macaques developed a febrile response, but duration and severity were indistinguishable from the controls (Fig 4B, Table 3). One Ly-IN macaque (Fig 3; animal 97−19) developed hypothermia in the recovery period but ultimately recovered. In the Lq-IV only group, no macaques developed a sustained febrile response like that seen in the controls or Ly-IN only groups. In the Ly-IN+Lq-IV group, one of the three macaques had a mild febrile response, but this response was substantially lower than that seen in the control or Ly-IN only groups (Fig 3). Although the control group was not significantly different from any other group in febrile response, likely due to the single control animal (Fig 3, animal 101−19) that did not exhibit a strong febrile response, the Ly-IN only group showed significantly higher fever severity than the Lq-IV and Ly-IN+Lq-IV groups during the first and second febrile phase (Table 3) (p < 0.05; two-way ANOVA).

### Serum viremia and viral RNA

**Fig 5:**
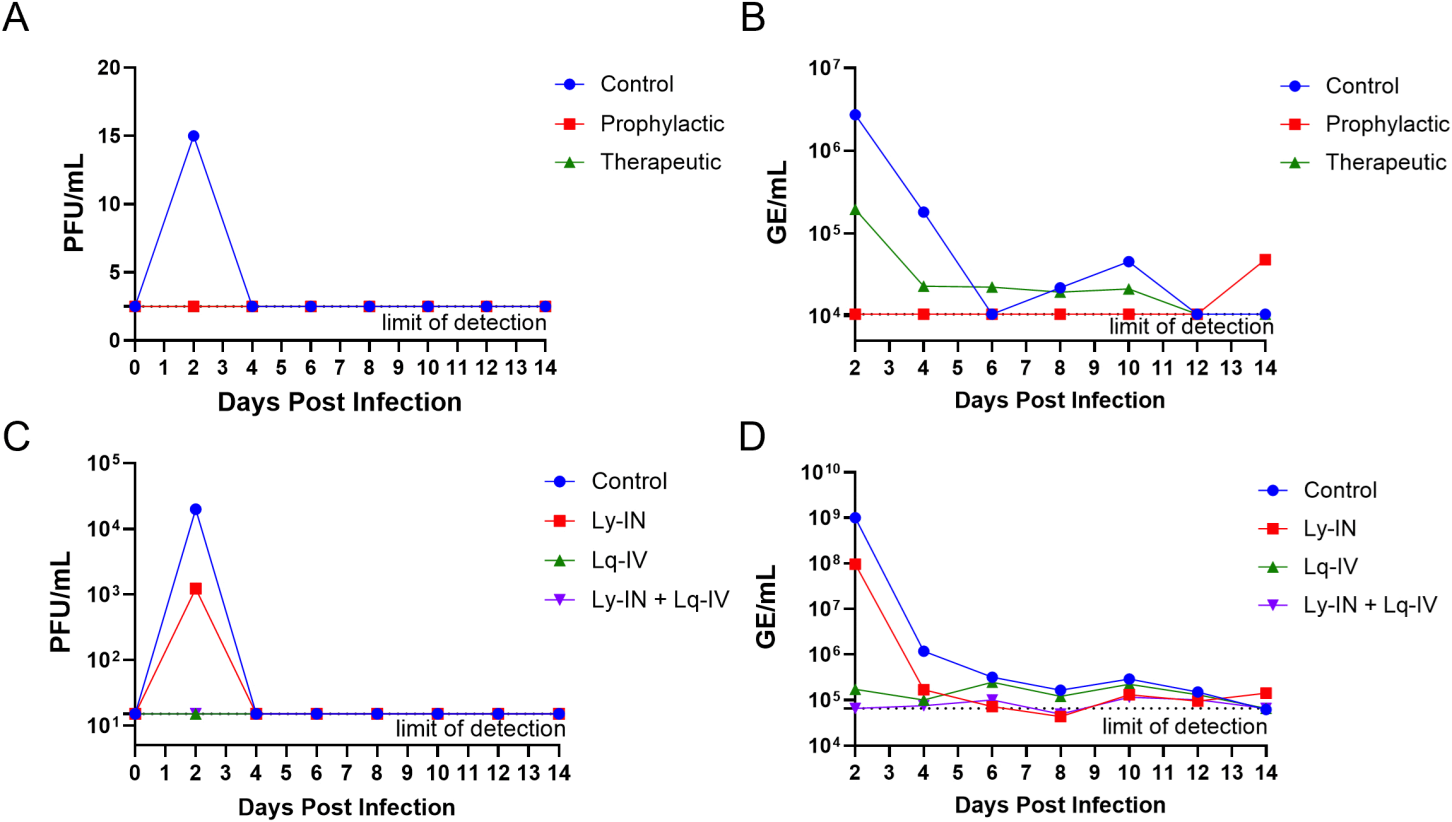
Viral burden in macaques. Serum plaque (A) and qRT-PCR genome equivalent (B) titers for cohort 1 macaques. Serum plaque (C) and qRT-PCR genome equivalent (D) titers for cohort 2 macaques. Median values for plaque titers and median geometric mean genome equivalent (GE) titers for qRT-PCR are shown. Error bars are omitted for clarity.

To monitor virus replication longitudinally, we quantified viremia via plaque assay and viral RNA via qRT-PCR using serum obtained from infected macaques. In cohort 1, low levels of infectious virus (VEEV TrD) were detected in control animals at 2 dpi (Fig 5A and Table 4). None of the treated animals exhibited detectable infectious virus at any time sampled after infection. Similarly, viral RNA was detected at high levels in control (Fig 5B and Table 5) but not in pre-treated animals. In parallel to viremia results, the control group exhibited the highest viral RNA on 2 dpi, which fell to undetectable levels by day 6. Due to variability between individual animals and small sample sizes, differences in serum viral RNA were not significant. The lack of viremia in the post-treatment group may have been due to treatment being administered on 1 dpi but the first post-infection sampling was on 2 dpi, leading to neutralization of infectious virions in circulation by 2 dpi. This is supported by the observation that viral RNA was recovered up to 10 dpi in the post-treatment therapeutic group, indicative of early virus replication and spread.

**Table 4:** Detection of infectious virus in sera of cohort 1 and cohort 2 macaques treated with irrelevant or anti-VEEV TcpAb preparations either before or after aerosol infection with VEEV.

Animal number	Day 0	Day 2	Day 4	Day 6	Day 8	Day 10	Day 12	Day 14
Cohort 1	Viremia detected (LOD = 2.5 PFU/mL)							
1 (control Lq-IV + Lq-IN)	–	+	–	–	–	–	–	–
2 (control Lq-IV + Lq-IN)	–	+	+	–	–	–	–	–
3 (control Lq-IV + Lq-IN)	–	+	–	–	–	–	–	–
4 (Pre. Lq-IV + Lq-IN)	–	–	–	–	–	–	–	–
5 (Pre. Lq-IV + Lq-IN)	–	–	–	–	–	–	–	–
6 (Pre. Lq-IV + Lq-IN)	–	–	–	–	–	–	–	–
7 (Post. Lq-IV + Lq-IN)	–	–	–	–	–	–	–	–
8 (Post. Lq-IV + Lq-IN)	–	–	–	–	–	–	–	–
9 (Post. Lq-IV + Lq-IN)	–	–	–	–	–	–	–	–
Cohort 2	Viremia detected (LOD = 25 PFU/mL)							
10 (control Ly-IN)	–	+	–	–	–	–	–	–
11 (control Ly-IN)	–	+	–	–	–	–	–	–
12 (control Ly-IN)	–	+	+	–	–	–	–	–
13 (Ly-IN)	–	+	–	–	–	–	–	–
14 (Ly-IN)	–	+	–	–	–	–	–	–
15 (Ly-IN)	–	+	–	–	–	–	–	–
16 (Ly-IN)	–	+	–	–	–	–	–	–
17 (Ly-IN)	–	+	–	–	–	–	–	–
18 (Lq-IV)	–	–	–	–	–	–	–	–
19 (Lq-IV)	–	–	–	–	–	–	–	–
20 (Lq-IV)	–	–	–	–	–	–	–	–
21 (Ly-IN+Lq-IV)	–	–	–	–	–	–	–	–
22 (Ly-IN+Lq-IV)	–	–	+^1^	+^1^	–	–	–	–
23 (Ly-IN+Lq-IV)	–	–	–	–	–	–	–	–

1. At the LOD of the assay (25 PFU/mL).

**Table 5:** Quantitation of viral genomes detected by qRT-PCT in sera of cohort 1 and cohort 2 macaques treated with irrelevant or anti-VEEV TcpAb preparations either before or after aerosol infection with VEEV.

Animal number	Day 0	Day 2	Day 4	Day 6	Day 8	Day 10	Day 12	Day 14
Cohort 1	LOD = 1.05x10^4^ GE/mL							
1 (control Lq-IV + Lq-IN)	–	6.45e6	1.05e4	1.05e4	1.05e4	8.58e5	1.05e4	1.05e4
2 (control Lq-IV + Lq-IN)	–	4.50e7	5.09e6	1.05e4	1.05e4	1.05e4	1.05e4	1.05e4
3 (control Lq-IV + Lq-IN)	–	7.01e4	1.12e5	1.05e4	9.65e5	1.05e4	1.05e4	1.05e4
4 (Pre. Lq-IV + Lq-IN)	–	1.05e4	1.05e4	1.05e4	1.05e4	1.05e4	1.05e4	1.05e4
5 (Pre. Lq-IV + Lq-IN)	–	1.05e4	1.05e4	1.05e4	1.05e4	1.05e4	1.05e4	1.05e4
6 (Pre. Lq-IV + Lq-IN)	–	1.05e4	1.05e4	1.05e4	1.05e4	1.05e4	1.05e4	1.05e4
7 (Post. Lq-IV + Lq-IN)	–	1.05e4	1.05e4	1.05e4	6.68e4	1.05e4	1.05e4	1.05e4
8 (Post. Lq-IV + Lq-IN)	–	2.70e7	1.10e5	1.01e5	1.05e4	8.77e4	1.05e4	1.05e4
9 (Post. Lq-IV + Lq-IN)	–	1.05e4	1.05e4	1.05e4	1.05e4	1.05e4	1.05e4	1.05e4
Cohort 2	LOD = 1.05x10^5^ GE/mL							
10 (control Ly-IN)	–	6.77e8	4.63e6	1.69e6	1.05e5	1.05e5	2.91e6	5.11e5
11 (control Ly-IN)	–	1.51e9	6.87e5	2.04e5	6.91e5	9.61e6	1.05e5	4.66e5
12 (control Ly-IN)	–	6.34e10	5.14e7	4.12e6	2.56e6	6.93e5	1.05e5	1.05e5
13 (Ly-IN)	–	1.53e8	4.65e6	2.56e6	1.05e5	7.80e5	1.59e6	2.19e6
14 (Ly-IN)	–	5.21e7	1.05e5	1.23e6	1.05e5	1.05e5	1.05e5	1.05e5
15 (Ly-IN)	–	8.10e8	2.93e6	1.05e5	1.05e5	3.00e6	1.05e5	1.12e6
16 (Ly-IN)	–	5.52e8	2.82e6	5.14e6	5.65e5	1.05e5	1.05e5	1.05e5
17 (Ly-IN)	–	2.36e9	1.05e5	1.39e5	1.05e5	1.05e5	1.05e5	1.05e5
18 (Lq-IV)	–	1.05e5	2.38e6	1.05e5	1.44e6	1.05e5	1.93e6	1.05e5
19 (Lq-IV)	–	1.05e5	1.05e5	1.05e5	1.05e5	1.05e5	1.05e5	1.05e5
20 (Lq-IV)	–	4.64e6	1.05e5	1.38e7	1.05e5	9.92e6	1.05e5	1.05e5
21 (Ly-IN+Lq-IV)	–	1.05e5	1.05e5	1.05e5	1.05e5	1.05e5	1.05e5	1.05e5
22 (Ly-IN+Lq-IV)	–	1.05e5	5.41e6	1.05e5	1.05e5	1.05e5	1.05e5	1.05e5
23 (Ly-IN+Lq-IV)	–	1.05e5	1.05e5	1.03e6	1.82e5	1.50e6	1.05e6	1.05e5
		At LOD or <10-fold above		>10-fold, < 100-fold above LOD		>100-fold above LOD		

In cohort 2, both control and Ly-IN groups were viremic for VEEV INH9813-K3E in serum on 2 dpi (Fig 5C and Table 4). In contrast, Lq-IV and Ly-IN+Lq-IV animals had limited to no detectable infectious virus in sera, suggesting these treatments significantly reduced viremia (p < 0.05, Kruskal-Wallis test). Despite this, one Ly-IN+Lq-IV animal showed serum viremia at the limit of detection on days 4 and 6 (Table 4). No difference in viremia was observed between the control and Ly-IN group. Similar to viremia, high titers of serum viral RNA were recovered in the control and Ly-IN groups, peaking on 2 dpi, with the control group showing 1.69 x 10^8^ to 1.59 x 10^10^ GE/mL of viral RNA (Fig 5D and Table 5). Little viral RNA was detected in serum of the Lq-IV and Ly-IN+Lq-IV groups, ranging from undetectable to 4.64 x 10^6^ GE/mL on 2 dpi, showing a significant reduction in serum viral RNA versus control (p < 0.05, Kruskal-Wallis test). It should be noted that the animal in the Ly-IN group that received half the intended dose of Ab (animal 97–19) exhibited the second lowest titers of the Ly-IN group, suggesting that the lower dose given to it did not result in higher virus titers. Overall, macaques receiving the IV formulation of SAB-131 were largely protected from serum viremia.

Interestingly, sporadic detection of viral genomes in sera occurred in some animals from all groups through 14 days post challenge and titers in some animals dropped below the limit of detection (LOD) but returned to detectable levels (Fig 5 and Table 5). For animals with detectable titers, all groups exhibited similar levels of viral RNA after 10 days post challenge. This type of persistent or spiking RNA profile has been observed previously in untreated macaques infected with INH9813-K3E [28] and any differences between INH9813-K3E and TrD likely reflect the magnitude of the initial viremia.

### Complete Blood Counts (CBC)

**Fig 6:**
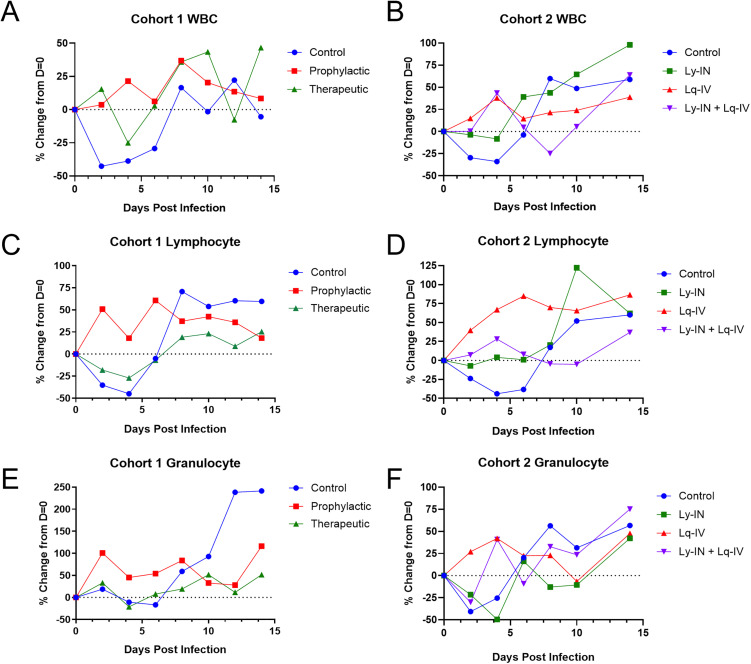
CBC analysis of VEEV-infected macaques. Median percent change from Day = 0 for white blood cell **(A,B)**, lymphocyte (C,D) and granulocyte (E,F) counts for cohort 1 (A, C, E) or cohort 2 (B, D, F) animals at two-day intervals after challenge (n = 3, except cohort 2 Ly-IN group of n = 5). Error bars are omitted for clarity.

VEEV-infected outbred macaques typically exhibit variable CBC results [28, 35, 36], and none of the current study data are significant by statistical analysis. However, several trends were evident (Fig 6). In cohort 1, pre-treatment animals did not exhibit lymphopenia observed in the control and post-treatment. While both post-treatment and control animals showed distinct lymphopenia, this began to resolve after SAB-131 post-treatment and possibly was less during clearance than controls. Granulocytes showed a similar pattern with the exception that the levels were higher in controls than either treatment group during the clearance phase, suggesting less inflammation in both treatment groups.

In cohort 2, leukopenia was observed on 2–6 dpi in control and Ly-IN groups with total WBC numbers decreasing when compared to pre-challenge levels (Fig 6B). Over a similar interval, Lq-IV and Ly-IN+Lq-IV groups exhibited an increase in WBC numbers, potentially indicative of a less severe response to infection. A similar pattern was observed with granulocytes.

### Plasma cytokines

**Fig 7:**
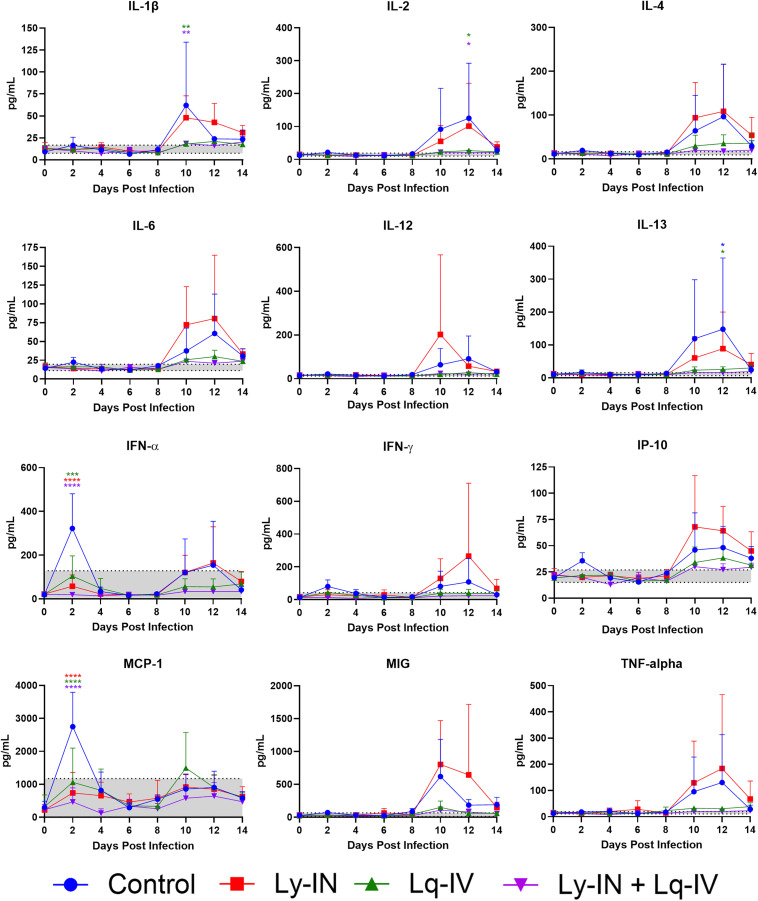
Cytokine induction in plasma from cohort 2 macaques. Data from multiple uninfected macaques [28] were used to determine a normal range as indicated by the shaded area bound by dotted lines. Significance differences, determined by two-way ANOVA with Dunnett’s post-test (*: p < 0.05, **: p < 0.01, ***: p < 0.001), are colored for each treatment group and compare each treatment group with the control group. Error bars are standard deviations and only upper bars are shown for clarity.

Cytokine quantification was only performed on cohort 2 animals. A number of measured cytokines demonstrated a peak at 2 dpi in the control, which showed either a significant (MCP-1, IFN-α) or a non-significant (IP-10, IFN-γ, IL-6) increase compared with Lq-IV, Ly-IN, and Lq-IV + Ly-IN groups (Fig 7). A number of cytokines also demonstrated increases in control and Ly-IN groups *versus* those receiving Lq-IV treatment at 8−14 dpi including IL-1β, IL-2, and IL-13 (Fig 7). Several of these cytokines are monocyte and T cell chemoattractant factors and/or pro-inflammatory mediators that we have reproducibly observed in the serum or brains of VEEV-infected mice and macaques after infection [28, 37]. This result is indicative of significant amelioration of this aspect of VEEV disease, which is typically attributed to the robust tropism of VEEV for myeloid cells present in lymphoid tissues [28,38]. Levels of some early cytokines, such as MCP-1 and IFN-α, were similar in all three treatment groups (Ly-IN only, Lq-IV and Ly-IN+Lq-IV groups) but lower than the control group (Fig 7). In addition, at 2 dpi, levels of IP-10, MCP-1, IFN-γ and IFN-α in control group sera were significantly higher than baseline at 0 dpi while the other treatment groups were not (S1 Fig). It had previously been assumed that serum proinflammatory cytokine levels were directly linked to the initial phase of the febrile response; however, since the Ly-IN only groups did not exhibit reduction in the first febrile phase (or any other measurement of disease), this assumption may be incorrect. We infer that the Ly-IN only treatment had an effect upon an aspect of virus replication that results in induction of some proinflammatory mediators as measured in serum. Further investigation of the relationship of IN treatment with lyophilized anti-VEEV Ab to serum cytokine levels is warranted.

### Histopathology

**Fig 8:**
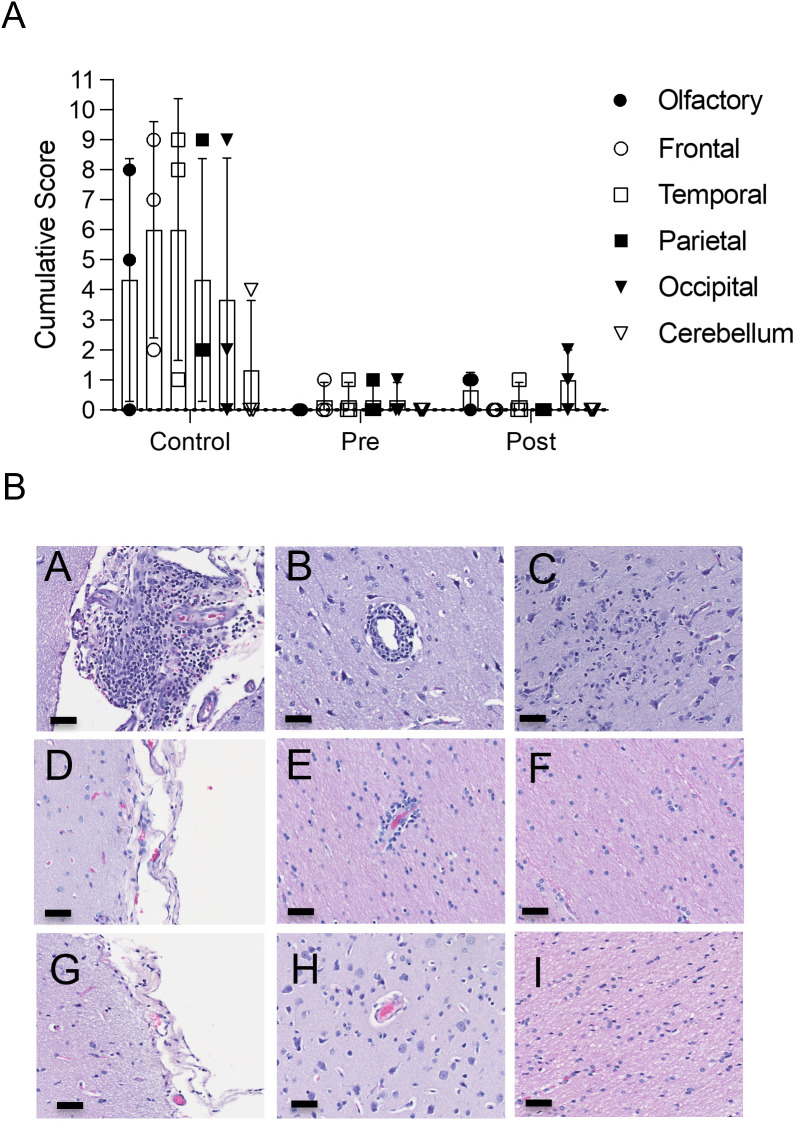
Histological analysis of cohort 1 macaques. **(A)** Histopathology ordinal scores for the six distinct anatomical regions of the brain from cohort 1 animals (n = 3/treatment). All animals were euthanized at 28 dpi. Hematoxylin and eosin (H&E) stained sections were generated blinded to study conditions. **(B)** Representative photomicrographs of H&E sections from the frontal lobes of VEEV TrD-infected control **(A-C)**, pre-treated (D-F) and post-treated (G-I) cohort 1 macaques. Scale bars = 50μm.

We examined histopathological changes in cohort 1. At 28 dpi, following the resolution of the acute phase of disease, tissues from six distinct anatomical regions of the brain were submitted for histopathology analysis to determine the severity of acute disease as well as the extent of long-term disease sequelae (Fig 8). The highest mean ordinal pathology scores were observed in the control group animals receiving anti-DENV2 antibodies (Fig 8A), which ranged qualitatively from mild to regionally marked mononuclear meningoencephalitis, glial nodules, rare neuronal satellitosis, and mononuclear perivascular cuffing (Fig 8B). Although lesions were observed in all anatomical locations of controls (cerebellum, olfactory bulb, and frontal, parietal, temporal, and occipital lobes), the highest mean scores were observed in the frontal and temporal lobes, with reduced severity of the occipital lobe and cerebellum (Fig 8A). Notably, vasculitis, overt neuronal degeneration and necrosis, and neutrophilic infiltration were not observed as has been observed with other alphaviruses in NHP models (i.e., eastern equine encephalitis and western equine encephalitis) [30, 39, 40]. In contrast, brain tissues from both VEEV Ab-treated groups were either histologically devoid of inflammation or contained minimal sporadic mononuclear meningeal and/or perivascular infiltrates, which has previously been reported as a normal incidental background findings in cynomolgus monkeys, and thus cannot be definitely attributed to VEEV [41]. Taken together, pathology results support the efficacy of the VEEV polyclonal TcpAb in both pre- and post-treatment platforms, with no discernible difference between the two approaches.

## Discussion

Aspects of the studies that inhibited our ability to assess efficacy of the TcpAb treatments include the small numbers of macaques in each treatment group, which limited the statistical analyses that could be performed for different disease biomarkers. However, in cohort 1, both the pre- and post-treatment experimental arms showed substantial protective effects on febrile responses and virus titers in serum, suggesting efficacy in reducing virus replication and disease severity. In cohort 2, prophylactic IV treatment alone or when combined with IN treatment also showed similar reductions in both parameters. Furthermore, in both cohorts, prototypical signs of VEEV disease in the blood such as lymphopenia exhibited clear trends towards protective effects of the TcpAb treatments. Histopathological analysis of cohort 1 animals showed a clear improvement of long-term central nervous system (CNS) lesions associated with the prophylactic and therapeutic IV treatments. Considering the small sample sizes, our results are proof-of-concept that IV anti-VEEV TcpAbs can be effective at ameliorating VEEV disease severity in both the pre- and post-treatment contexts. Pre-treatment reduced the severity of all aspects of disease while post-treatment was effective at reducing the second febrile peak and associated disease signs as well as the duration of illness. Further studies with larger group sizes and additional virological and host response measurements (e.g., tissue virus titration, presence of persistent virus genomes in CNS, intracranial pressure, electroencephalogram, electrocardiogram) are clearly warranted [28, 32, 42, 43]. The fact that disease symptoms and virus titers could be affected by treatment at 24 hours post-exposure in cohort 1 is especially encouraging regarding the use of TcpAb preparations for post-exposure therapy, especially when similar results were observed in other TcpAb models [19].

In terms of formulation, only liquid intravenous SAB-131 treatment has a measurable ameliorative effect on multiple parameters of VEEV disease when compared with Ly-IN antibody treatment in the cynomolgus macaque model. In cohort 2, significant reduction in virus plaque titers on all sampled days and viral RNA on 2 dpi were observed in the Lq-IV and Lq-IN+Ly-IV groups, as were reductions in serum proinflammatory cytokine levels and fever in both the first and the second febrile phases. In addition, IV treatments largely ameliorated a second cytokine induction peak between 8 and 12 dpi that had not been previously detected after VEEV infection (most likely due to omission of these times from prior study cytokine sampling schemes). CBC results suggest an appropriate proliferative WBC response to mild infection as opposed to a pathological leukopenia observed in control and Ly-IN only groups.

Regarding differences in efficacy of SAB-131 between TrD and INH9813-K3E, the original immunogen for bovine immunization was inactivated TrD derived from a cDNA clone, essentially identical to the cohort 1 challenge virus. Other studies have shown cross-reactive neutralizing epitopes between IA/B and IC clade viruses and Old and New World alphaviruses [44–46], so we expected some degree of cross protection. Effects on disease manifestations for both viruses were similar, although, the study design precluded a detailed statistical analysis. Ultimately, it is likely that the polyclonal nature of the anti-VEEV TcpAb overcame differences that might exist in specific neutralizing/therapeutic epitopes. The most striking differences between the TrD and INH9813-K3E infections was in macaques receiving control antibodies, where TrD exhibited several orders of magnitude lower serum infectious viremia and genome than in INH9813-K3E. This result is consistent with our previous reports of macaque infection with TrD and INH9813-K3E [28]. Mechanisms underlying these observations are deserving of further study.

Because primary effects of epizootic VEEV infection in most human symptomatic cases are comprised of pyrexia, malaise, myositis, photophobia, and headache, it is possible that treatment with SAB-131 intravenously would reduce these effects to the point where human productivity may not be highly impacted. Because the current macaque model is non-lethal, our results may not apply to the most severe manifestations of disease, which can result in mortality in a small percentage of VEEV-infected humans (reviewed in [47]).

Our studies have several limitations. While we observed that Ly-IN treatment alone did not reduce any measured disease parameter when compared to control antibody, except for several serum cytokines on 2 dpi, and additional IN treatment with lyophilized antibody did not further reduce virus replication or disease severity when combined with IV treatments, our result does not necessary rule out a potential effect of a Ly-IN treatment. A more detailed study on antibody distribution in serum and respiratory systems after Ly-IN delivery will be needed, coupled with direct comparison against a control antibody delivered in a Ly-IN formulation. Testing of different IN delivery devices may also address potential technical challenges of delivering a lyophilized antibody. Furthermore, a 100 mg/kg Lq-IV dose of antibody may be too high and have masked any potential synergistic protective effect brought by Ly-IN formulation, as a Lq-IV dose of 50 mg/kg has been demonstrated to be safe and/or effective against various viruses in murine or human studies [15,17,18,48]. Given the current lack of understanding of the route of VEEV migration from aerosol delivery sites to the central nervous system in primates, it may be prudent to test delivery of antiviral antibody preparations deeper into the lungs of experimental animals in case a pulmonary-hematogenous route of CNS entry is important to CNS disease.

## Supporting information

S1 Fig Early plasma cytokine levels from cohort 2 macaques. Data were derived from Fig 7 for cytokines where the control group was significantly greater than anti-VEEV TcpAb-treated animals. Differences between treatment groups were analyzed by two-way ANOVA with Dunnett’s post-hoc test comparing to 0 dpi (*: p < 0.05, **: p < 0.01, ***: p < 0.001). Shaded areas bound by dotted lines indicate the range of values in uninfected macaques from reference [28]. Error bars are standard deviations.
(TIF)

S1 Table Clinical scoring parameter. (DOCX)

S1 Data Raw data for figures. (XLSX)
