# Supplementary material for: A single dose of purified human antibody from transchromosomic bovines mitigates aerosolized Venezuelan equine encephalitis virus disease in cynomolgus macaques
Source: PLoS One. 2026 Apr 27;21(4):e0347864. doi: 10.1371/journal.pone.0347864 (PMC13120304; doi:10.1371/journal.pone.0347864)
Supplement: S1 Table — (DOCX) [file pone.0347864.s002.docx]

**S1 Table. Clinical scoring parameter.**

| Neurologic score | Description |
| --- | --- |
| 1 | Normal |
| 2 | Mild photophobia, muscle stiffness, jaw soreness/stiffness |
| 3 | Head pressing, frequent loss of balance, tremors or rigors, moderate photophobia, frequently swatting at head |
| 4 | Seizure activity |
| 5 | complete loss of balance or comatose, prompt euthanasia |
| Activity score | Description |
| 1 | Normal (frequent eye contact and reaction) |
| 2 | Less active (respond to observer, but less frequent or intense interaction) |
| 3 | Sluggish (only respond when prodded, hunched posture, limited interaction) |
| 4 | Upright but inactive (ignored observer or stimuli) |
| 5 | Does not respond/moribund, prompt euthanasia |
| Temperature score | Description |
| 1 | Normal (baseline to 1.5 degrees Celsius above baseline) |
| 2 | Mild fever (1.6 to 3.0 degrees above baseline) |
| 3 | Moderate fever (3.1 to 4.0 degrees above baseline) |
| 4 | Severe fever (greater than 4.0 degrees above baseline) |
| 5 | Moderate hypothermia (2.1 to 5 degrees below baseline) |
| 6 | Sever hypothermia (greater than 5.0 degrees below baseline), prompt euthanasia |
